# Influence of simulated microgravity on mechanical properties in the human triceps surae muscle in vivo. I: Effect of 120 days of bed-rest without physical training on human muscle musculo-tendinous stiffness and contractile properties in young women

**DOI:** 10.1007/s00421-014-2818-9

**Published:** 2014-02-08

**Authors:** Yuri A. Koryak

**Affiliations:** SSC, Institute of Biomedical Problems RAS, 76-A Khoroshevskoye Shosse, 123007 Moscow, Russia

**Keywords:** Bed-rest, Triceps surae muscle, Electromechanical delay, Musculo-tendinous stiffness, Contractile properties

## Abstract

**Purpose:**

The aim of this study was to investigate the effect of a 120-day 5° head-down tilt (HDT) bed-rest on the mechanical properties of the human triceps surae muscle in healthy young women subjects.

**Methods:**

Measurements included examination of the properties of maximal voluntary contractions (MVC), twitch contractions (*P*_t_) and tetanic contractions (*P*_o_). The difference between *P*_o_ and MVC expressed as a percentage of *P*_o_ and referred to as force deficiency (*P*_d_), was calculated. Electromyographic (EMG) activity in the soleus muscle, electromechanical delay (EMD) and total reaction time (TRT) were also calculated. EMD was the time interval between the change in EMG and the onset of muscle tension. Premotor time (PMT) was taken to be the time interval from the delivery of the signal to change in EMG.

**Results:**

After HDT *P*_t_, MVC and *P*_o_ had decreased by 11.5, 36.1, 24.4 %, respectively, *P*_d_ had increased by 38.8 %. Time-to-peak tension had increased by 13.6 %, but half-relaxation time had decreased by 19.2 %. The rate of rise in isometric voluntary tension development had reduced, but no changes were observed in the electrically evoked contraction. EMD had increased by 27.4 %; PMT and TRT decreased by 21.4, and 13.7 %, respectively.

**Conclusion:**

The experimental findings indicated that neural as well as muscle adaptation occurred in response to HDT. EMD is a simple and quick method for evaluation of muscle stiffness changes and can serve as an indicator of the functional condition of the neuromuscular system.

## Introduction

A number of studies have documented that the microgravity environment encountered during space flight or simulated by using models of weightlessness induces alterations in skeletal muscle function (Fitts et al. 2000, 2010; di Prampero and Narici 2003; Gopalakrishnan et al. 2010). The phenomenon of decrease in functions and working capacity of muscles after the long period of unloading of the muscular device is usually interpreted as result of lack of gravitational loading. Influence of mechanical unloading on functions and working capacity of skeletal muscles on a person has been extensively investigated (Edgerton and Roy 2000; di Prampero and Narici 2003; Rittweger et al. 2006, 2013; Reeves et al. 2005; Mulder et al. 2008; Miokovic et al. 2011). It has been shown that, as a consequence of reduction in activity, muscular atrophy preferentially occurs in antigravity muscles (Thomason and Booth 1990). It is shown, that the exposure of the human to conditions of the lowered muscular activity (a condition of 0 G) is accompanied by development of progressive “weakness”. “Weakness” of muscles is reflected in registered mechanical properties. Cosmonauts/astronauts are examples where both fitness and performance levels may decline during missions. Various studies have reported decrements in muscle mass/volume, strength, power, and endurance performance after short-term space flights (Akima et al. 2000; di Prampero and Narici 2003; Tesch et al. 2005; koryak et al. 2007; Trappe et al. 2009; Fitts et al. 2010; Gopalakrishnan et al. 2010; Koryak 2001, 2011a, b; Koryak et al. 2007). Measurements of crewmembers who returned from missions on the Mir Station have found changes of 12–20 % in volume (Zange et al. 1997), up to a 48 % decline in maximal voluntary contraction (strength) of the plantar flexor muscles (Zange et al. 1997), and decrease in velocity contraction the plantar flexor muscles of ~8 % (Koryak 2001, 2011a). Knee extensor and flexor endurance measured as total work performed was found to decrease by about 26 % in two crewmembers on the International Space Station after space flight (Lee et al. 2000).

In the absence of weight-bearing activity, strength loss is the most evident consequence of atrophy. This is also reflected by changes in fiber size and/or fiber type (Widrick et al. 1999). For instance, many studies indicate a relative increase in fast-twitch fibers in slow contraction muscle (Fitts et al. 2000; Trappe et al. 2004). Trappe et al. (2004) found directional shift from slower contracting fiber to a faster contracting fiber. This fiber-type transition phenomenon also affects muscle mechanical properties, leading to an increase in shortening velocity (Thomason and Booth 1990; Edgerton and Roy 1996) and a decrease in stiffness (Canon and Goubel 1995; Goubel 1997).

Loss of muscle mass, and force, and neuromuscular performance, has been reported after spaceflight or prolonged bed-rest, whereas the velocity characteristics measured in muscle groups were not always significantly modified (Grigoŕyeva and Kozlovskaya 1987; Dudley et al. 1992; Koryak 1995, 2001, 2002). It was demonstrated that the unloaded shortening velocity measured in single human soleus fibers shifted toward higher velocities after simulated or real microgravity (Widrick et al. 1998, 1999). Moreover, change of amplitude electromyography (EMG) and relationship force/EMG, showed, that the nervous system subjects reorganization a pattern recruitment motor units with their displacement aside recruitment fast motor unit against slow (Recktenwald et al. 1999).

Surface EMG data show the electrical activity of muscle and used in the analysis of human movement. It is well known that there is a delay between the onset of active state in skeletal muscle and the development of tension. This delay, called here electromechanical delay (EMD), was important in the formulation of the two-component model of muscle by Hill (1950) in which he postulated that the slow development of tension was due to the presence of elastic elements in series with the contractile element. EMD is a measure of the time lag between muscle activation and muscle force production (Cavanagh and Komi 1979; Viitasalo and Komi 1981). Thus, EMD is primarily a measure of series elastic stiffness. Stiffness describes the relation between force and stretch length. A mechanically stiff muscle will transmit large forces with very little stretch of the series elastic components. Conversely, a mechanically compliant or lax tissue requires much greater muscle contraction to sufficiently stretch the elastic components and generate measurable force. Compliant tissues require more time from activation until force generation, i.e., their EMD is longer.

Exposure of humans to zero gravity has been reported to induce a progressive weakness of the antigravity skeletal muscles. Muscle atrophy will remain a risk, particularly during longer missions by cosmonauts and astronauts for the construction and operation of a space station, or during a voyage to another planet.

Although the deterioration of musculoskeletal function may not present an immediate health or operational hazard during short-term flight after space missions of long duration if not counteracted these effects of microgravity can become serious problems upon return to Earth. Therefore, measures designed to maintain the effective functioning of all body systems during weightlessness, as if still under the influence of the gravitational field of the Earth, are extremely important.

Data of influence of unloading on mechanical properties of muscles of women in the literature are not present. This is the first study to make quantitative measurements of the functional properties and EMD, and musculo-tendinous stiffness (MTS) of a single muscle in young women exposed to long-term bed-rest without countermeasures. The present study was designed to investigate the effects of a long controlled period of voluntary bed-rest (simulated microgravity) on the electrically evoked and voluntary mechanical properties of the muscles (the triceps surae—TS) of the lower leg in normal, healthy subjects (young women). Thus, the first aim of the present study was to investigate changes in force, and velocity, and force–velocity characteristics in human muscles as a result of a long-term bed-rest (120 days).

Muscle and joint stiffness are important parameters for movement control because their value determines the resistance to an external perturbation. Furthermore, muscle stiffness can be modulated through changes in neural activation (Kirsch et al. 1994). The literature indicates that disuse induces an increase in muscle and joint stiffness and a decrease in the range of motion (Akeson et al. 1987; Lebiedowska and Fisk 1999; Lambertz et al. 2001, 2003; Grosset et al. 2010). This may make normal movement more difficult and may alter neuromuscular performance, because stiffness governs the mechanics of the interaction between the musculoskeletal system and the external environment. If such changes occur during spaceflight, daily work in a space station could become critical. Therefore, the second purpose of the present work was to determine MTS of human the TS and changes in her after a long-term bed-rest.

In the current study, we report changes in contractile properties of skeletal muscle in four crewmembers after a 120-day 5° HDT without physical training. We also provide information on EMD and MTS. The unique aspect of this study is measurement of an EMD that can be an indirect index of degree of changes MTS of a muscle.

## Materials and methods

### Subjects

Four healthy active women volunteered to participate in this study. Their mean age, height and mass were 31.5 (SEM 1.7) years (range 28.0–36.0), 162.3 (SEM 1.9) (range 158.0–167.0) cm and 55.0 (SEM 1.8) (range 51.0–59.0) kg, respectively. They were given detailed information about the purpose of the study and methods used and gave written consent. None of the subjects had experiences low back pain.

Selection of subjects was based on an evaluation that consisted of taking a detailed medical history, and a physical examination, complete blood count, urinalysis, resting electrocardiogram, and a selection of blood chemistry analyses, which included the estimation of concentrations of fasting blood glucose, blood urea nitrogen, creatinine, lactic dehydrogenase, serum transaminase bilirubin, uric acid, and cholesterol. No subject was taking medication at the time of the study, and all the subjects were non-smokers and recreationally active, but not especially well trained. Each subject served as her own control.

This study was conducted according to the Helsinski Statement (1975) and has been approved by the local Ethics Committee in Moscow at the Institute of Biomedical Problems.

### Bed-rest

Bed-rest for 120 days in an antiorthostatic position (5° HDT) of the body was used as a model of the long-term hypokinesia/hypodynamia effect of space flight. The 5° HDT position was chosen since various physiological alterations induced by actual spaceflight have been shown to be similar to those reported in ground-based studies using this model (Sander and Vernikos 1986).

During this 120-day experiment, the subjects were housed 24 h day^−1^ in the Human Research Facility of the Health Ministry Institute of Biomedical Problems. During bed-rest, the subjects remained in the HDT position. They were allowed to use the toilet at any time, and a shower was given every 3 days. During transportation the subjects lay on a stretcher. The room temperature of the wards did not exceed 25 °C.

### Experimental setup

The subjects were carefully familiarized with the test procedures of voluntary force production during several warm-up contractions preceding the actual maximal contractions and were allowed to habituate to the electrical stimulation procedures during preliminary visits to the laboratory before definitive control measurements were taken. To ensure standardization of position and fixation of the limb during assessment, a special setup, previously shown by Koryak (1985), was used. The dynamometer and recording system used to measure the forces produced by electrical and voluntary contractions of the TS have previously been described in detail (Koryak 1985). In brief, the subject was seated comfortably on a special chair in a standard position (at a knee joint angle between the tibia and the sole of the foot of 90°). The position of the seat was adjusted to the individual and then firmly secured. A rigid leg fixation ensured isometric conditions for the muscle contraction. The dynamometer was a steel ring with a saddle-shaped block attached to fit the Achilles tendon. The resting pressure between the sensor and the tendon was constant for all the subjects and was set at 5 kg. The contractile properties of the TS were tested twice: 10–8 days before the beginning of the bed-rest and after it ended. The test protocol was identical for both prebed-rest and postbed-rest tests.

### Electrical stimulation

All the recordings were made in a room at constant temperature (22 ± 1 °C). The TS of the dominant limb was stimulated under isometric condition by a neuromuscular stimulator (model “ESU-1”, USSR). Electrical stimulation was applied through monopolar electrodes, one (the cathode) 1 cm in diameter, was located in the popliteal fossa (tibial nerve) which is the site of lowest resistance, and the other electrode (the anode) was positioned on the lower one-third of the front surface of the femur. Voltage was increased in stepwise manner until maximal twitch response was evoked. A single stimulus was given every 30 s.

### Electromyography recording

Bipolar surface electrodes (standard *Ag/AgCl* electrodes, 8 mm in diameter, spaced 25 mm center-to-center) were placed 6 cm below the insertion of the gastrocnemii on the Achilles tendon for the soleus. The ground electrode (7.5 × 6.5 cm silver plate) was placed over the tibia. The skin was rubbed with an abrasive paste and cleaned with alcohol to reduce the inter-electrode impedance to around 5 kΩ. Electrode gel was used with all surface electrodes.

### Procedure

Contractile properties of the human TS estimated on mechanical parameters voluntary and electrical (involuntary) contractions. The experimental protocol consisted of four parts.

1. Maximal voluntary contraction (MVC) was estimated according to the tendogram of isometric voluntary contraction performed on the instruction condition to exert maximal contraction. 2–3 maximal contractions were usually recorded from each subject until maximal force contractions was obtained. There was a 1–2 min rest between the sets. The MVC was determined as the highest value of voluntary force recorded during the entire contraction. The force was recorded on magnetic tape.
  The subjects were also carefully instructed to respond to an auditory signal by exerting MVC as rapidly as possible, and to maintain it as long as the signal was audible (~1.5–2.0 s). In the force–time curves, the times taken to increase the force to 25, 50, 75, and 90 % of MVC were calculated (Koryak 1985; Koryak 2011a).
  Involuntary (electrically evoked) isometric contraction (twitch contraction, double and tetanic) of the human TS is caused by electrical stimulation of the *tibial nerve*, using a neuromuscular stimulator.
  The isometric twitch and tetanic contractions of the TS were induced by electrical stimulation of the tibial nerve using supramaximal rectangular pulses of 1 ms duration. Maximal isometric twitch force (*P*_t_) was estimated according to the tendogram of the TS isometric twitch response to a single electrical stimulus applied to the tibial nerve (Fig. 1a, left panel). The maximal force (*P*_o_) was estimated by the tendogram from the evoked contraction in response to an electrical tetanic stimulation of the nerve, innervating the TS, with a frequency of 150 impulses s^−1^ (2) (Fig. 1a, right panel). The difference between *P*_o_ and MVC expressed as a percentage of the *P*_o_ value and referred to as force deficiency (*P*_d_) has also been calculated. This parameter reflects the capability of a certain part of the motor pool (Koryak 1985) (Fig. 1a, right panel). The smaller the *P*_d_, the more complete is central control over the muscle system when exerting MVC.

**Fig. 1:**
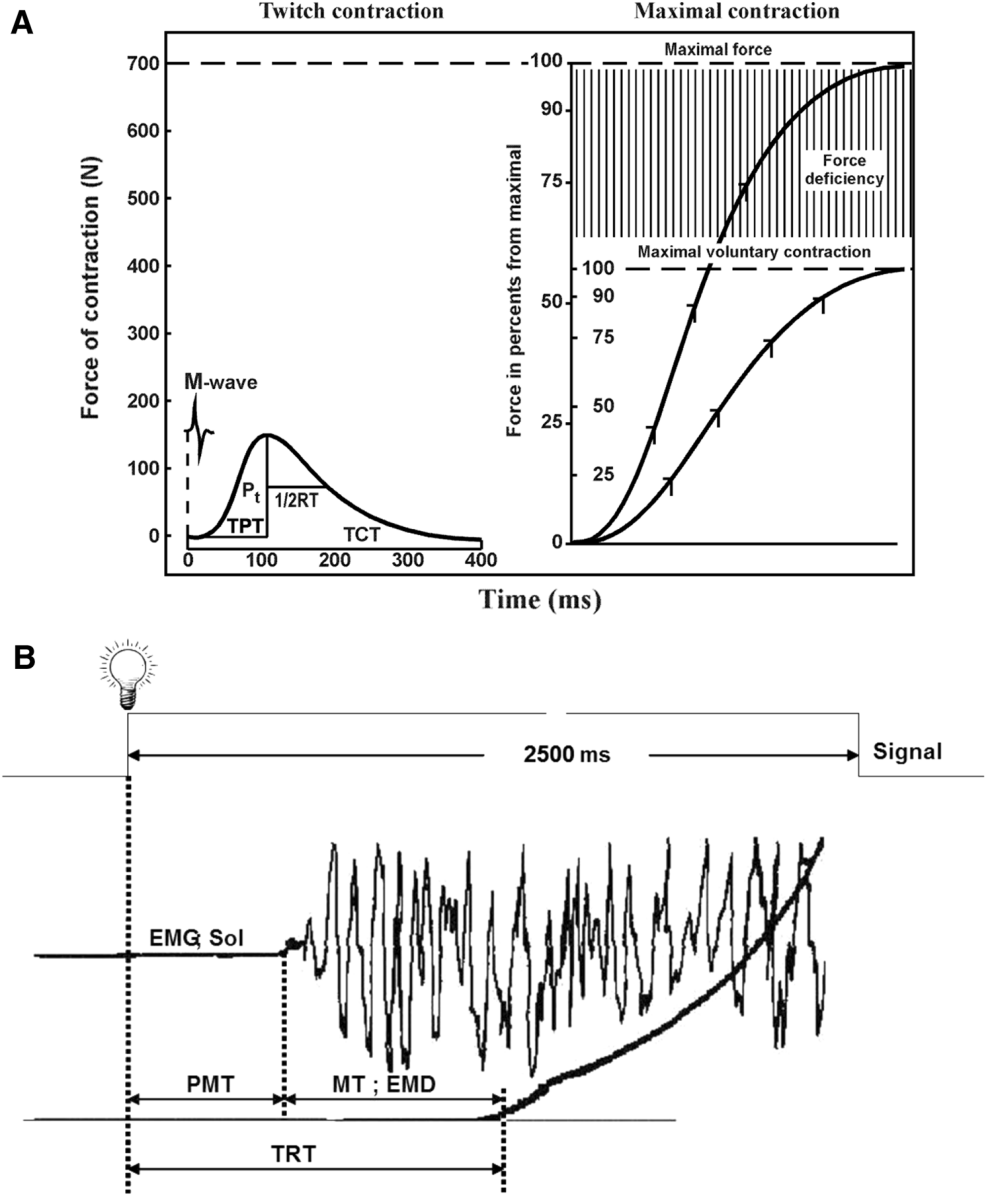
**a** Examples of isometric twitch contraction curves (*left panel*) and electrically evoked tetanic tension and voluntary muscle tension development (*right pane*l) showing how the parameters of the mechanical responses of muscle contraction were subsequently calculated. *TPT* time-to-peak tension, *1/2 RT* half-relaxation time, *TCT* total contraction time, *P*_t_ twitch force. **b** Schematic representation of a sample contraction showing total reaction time (TRT) with its premotor (PMT) and motor (MT) components, force–time curve and EMG recorded from m. soleus
  After a rest of 4–5 min, the motor nerve was stimulated at various intervals. Supramaximal twin stimuli at 330, 250, 200, 100, 50, and 20 impulses s^−1^ were applied (Koryak 1985; Koryak 2011a, b). The maximal strength (amplitude) of the muscle contraction was determined and expressed as a percentage of the twitch contraction.
  Tetanic index (TI) was expressed as the relation of *P*_o_/*P*_t_ (Close 1972; Koryak 1985).
2. The time from the moment of stimulation to peak twitch (TPT) and the time from contraction peak to half-relaxation (1/2 RT) were calculated by the tendogram of isometric twitch (Fig. 1a, left panel). The accuracy of measurement was 1 ms.
3. The subjects were also carefully instructed to respond to an auditory signal by exerting MVC as rapidly as possible, and to maintain it as long as the signal was audible (~1.5–2.0 s). In the force–time curves, the time taken to increase the force to 25, 50, 75, and 90 % of MVC was calculated (Koryak 1985). Similarly, the rate of rise of evoked contraction in response to electric stimulation of the nerve with a frequency of 150 impulses s^−1^ was determined (Koryak 1985) (Fig. 1a, right panel). The accuracy of measurement was 1 ms.
4. On a light signal the subject carried out plantar flexor under condition of “to contract as it is possible quickly and strongly” (Fig. 1b). Voluntary contraction in response to a visual stimulus (flash lamp) was adopted as a rapid ballistic movement. The signal to movement of “explosive” character was the visual diode—lamp (Ø 7 mm, 1 W)—was placed at eye level 1 m in front of the subject. The signals lasted 2.5 s and the pause between the signals was random ranging 1.4–5.0 s. The threshold for force was 5 N.
  A separate timer was used to record the time interval from the presentation of the light signal to movement. A special timer allowing synchrony with presentation of a light signal to the beginning of movement to record the development of mechanical answer of the human TS, was used.
  From the tendogram total reaction time (TRT), defined as the time interval from the application of the light stimulus to movement, was estimated. TPT was divided into pre-motor (PMT), defined as the time interval from the application of the stimulus to the change in electrical activity of the soleus muscle, and motor time (MT or electromechanical delay—EMD), defined as the time interval from the change in electrical activity in the soleus muscle to movement (Weiss 1965) (Fig. 1b). The force thresholds were also taken as relative values of 2 % from the maximum isometric force level of each contraction.
  Subjects were permitted 3 practice trials separated by 30 s and in most cases the mean of 3 readings was used to determine TRT, PMT and EMD.

### Statistical analysis

Conventional statistical methods were used for the calculation of means and standard errors of the mean. Differences between baseline (background) values of the subject and those post-exposure (bed-rest) were tested for significance by Student’s paired *t* test. Values are given as mean ± SEM throughout. Significant differences between means were set at the *p* < 0.05 level. The percentage changes for pre- and postbed-rest were calculated.

The strength of the relationship between EMD and MVC and between EMD and “*ballistic*” voluntary contraction was determined by Pearson’s coefficient of correlation.

## Results

**Fig. 2:**
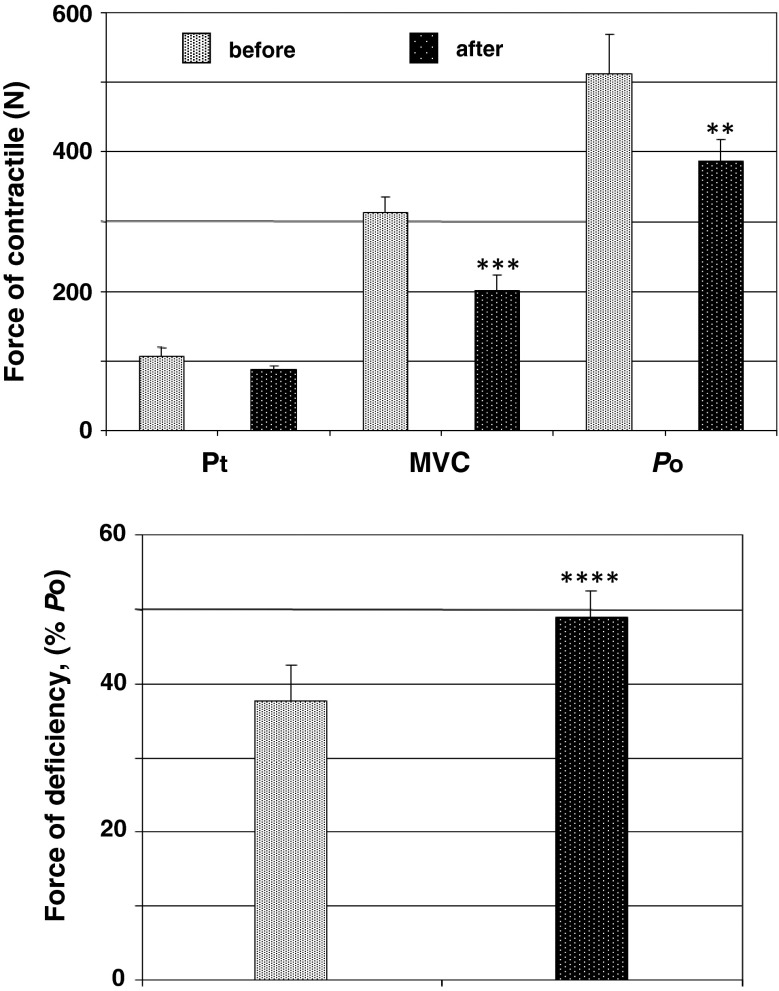
The effect of a 120-day 5° head-down tilt on the maximal twitch response of force (*P*_t_), maximal voluntary contraction (*MVC*), and evoked electrical tetanic stimulation at a frequency of 150 impulses s^−1^ (*P*_o_) (*top panel*) and force deficiency (*P*_d_) (*bottom panel*) of the triceps surae muscle. **p < 0.02; ***p < 0.01; ****p < 0.001

The mean changes in the TS tension under HDT are shown in Fig. 2 (top panel) and reveal a significant decrease. Isometric *P*_t_ decreased by a mean of 11.5 % [pre 105 (SEM 12.8) N compared to post 85.3 (SEM 5.9) N; *p* > 0.05], MVC by a mean of 36.1 % [pre 307.1 (SEM 21.6) N compared to post 196.2 (SEM 22.6) N; *p* < 0.01] N and *P*_o_ by a mean of 24.4 % [pre 503.3 (SEM 55.9) N compared to post 380.6 (SEM 28.4) N; *p* < 0.02]. The *P*_d_ increased significantly by a mean of 39.8 % [pre 37.6 (SEM 4.7)  % compared to post 48.8 (SEM 3.7)  %; *p* < 0.001] after HDT (cf. Fig. 2, bottom panel).

**Fig. 3:**
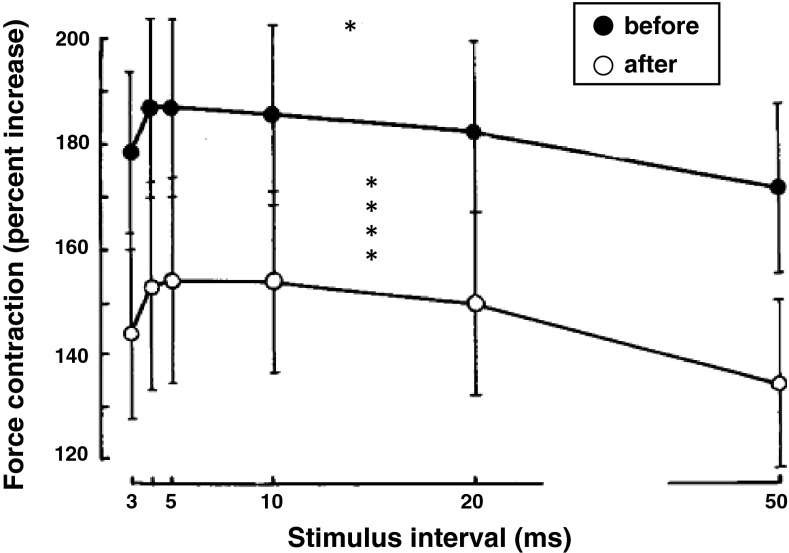
The effect of a 120-day 5° head-down tilt on the maximal force of contraction of triceps surae muscle with double stimulation with varying intervals between impulses. **p < 0.05; ****p < 0.001

Mean changes in isometric force of the TS under paired stimulation of maximal intensity when twin stimuli were applied at 3, 4, 5, 10, 20, 50 ms separation are presented in Fig. 3. The greatest force of contraction was observed at intervals of 4–10 ms and decreases or increases of interval from this range was accompanied by considerable decreases (*p* < 0.05) with no change in the general pattern of muscle tension developed. At any given interpulse interval the relative increase in force of contraction after 120-day HDT effect was significantly less compared to the control value (*p* < 0.001).

**Fig. 4:**
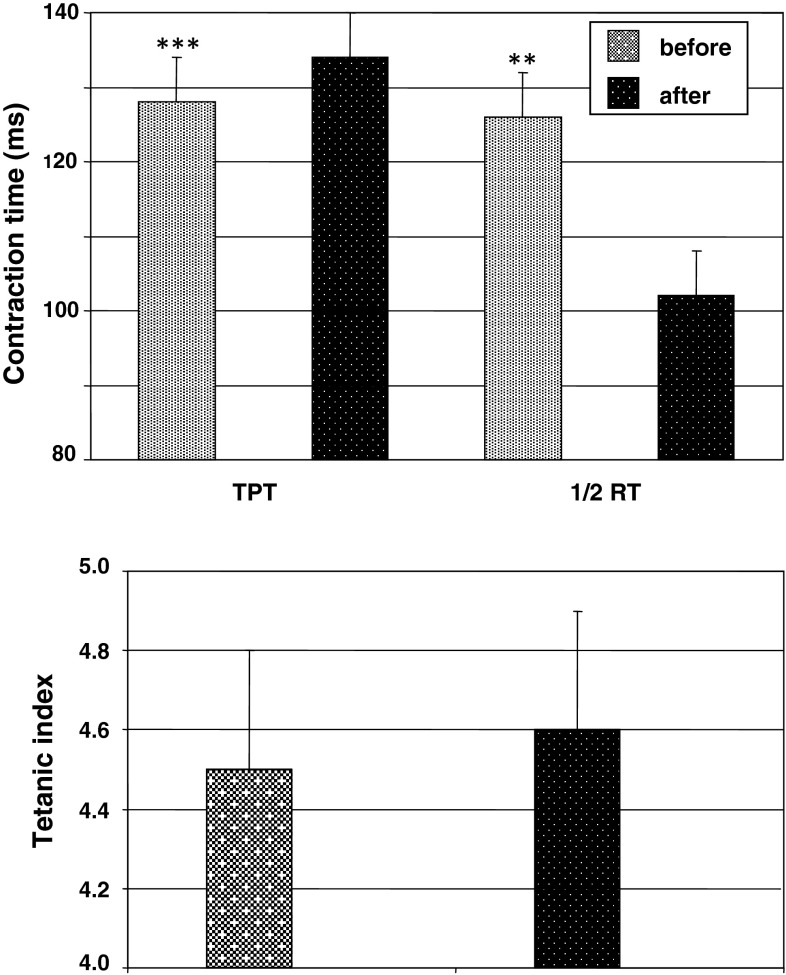
The effect of a 120-day 5° head-down tilt on the isometric twitch time-to-peak tension (TPT) and half-relaxation time (1/2 RT) (*top panel*), and tetanic index (*bottom panel*) of the triceps surae muscle. **p < 0.05; ***p < 0.01

The change in mean time of isometric twitch contraction as the opposite value to contraction velocity for the TS after a 120-day HDT effect is given in Fig. 4 (top panel). As is seen from the data analysis, exposure to HDT conditions was accompanied by a statistically significant decrease of the muscle contraction and increased relaxation velocity. Thus, TPT increased by a mean of 13.6 % [pre 118 (SEM 5) ms compared to post 134 (SEM 5) ms; *p* < 0.01], 1/2 RT decreased by a mean of 19.2 % [pre 123 (SEM 6) ms compared to post 102 (SEM 7) ms; *p* < 0.02].

TI increased by a mean of 4.2 % (*p* > 0.05) (Fig. 4, bottom panel).

**Fig. 5:**
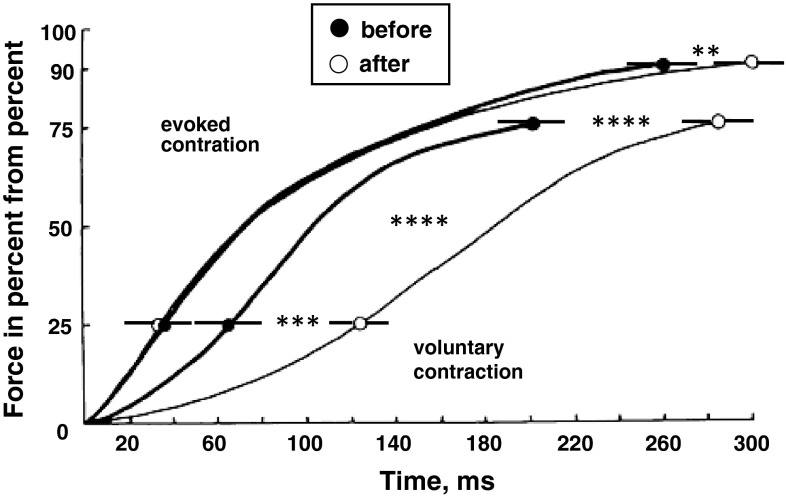
The effect of a 120-day 5° head-down tilt on the maximal rates of development of voluntary isometric force and of electrically evoked tetanus expressed both on relative scales. **p < 0.02; ***p < 0.01; ****p < 0.001

Mean changes in the rate of development of isometric tension of the TS are given in Fig. 5 (top panel). The analysis of the data gives evidence of a decrease in the rate of rise in isometric voluntary tension development of the TS (*p* < 0.01–0.001). This may be seen as a decrease in the convexity of the force–time curve estimated according to a relative scale. However, in the assessment of the force–velocity muscle properties, no substantial changes were observed on the effect of 120-day HDT on isometric electrically evoked tetanic development (*p* > 0.05).

**Fig. 6:**
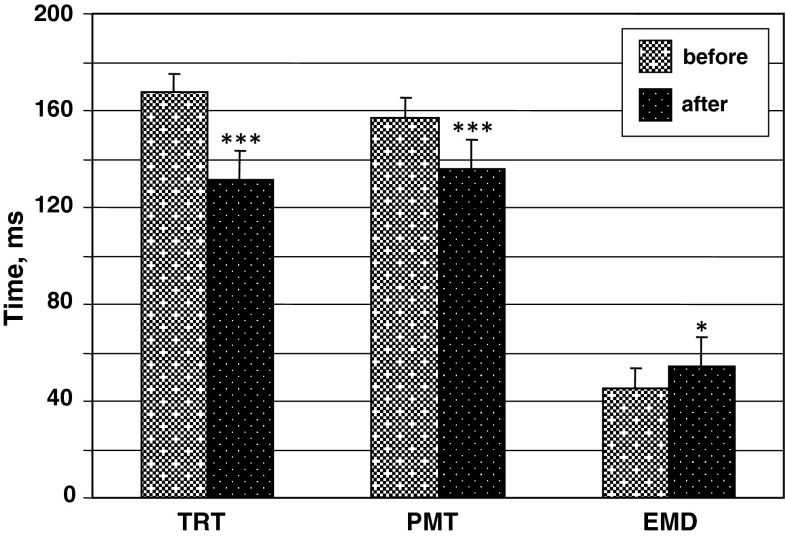
The effect of a 120-day 5° head-down tilt on total reaction time (*TRT*), premotor (*PMT*) and electromechanical delay (*EMD*) components. *p < 0.05; ***p < 0.01

The mean changes in EMD under HDT are shown in Fig. 6. The EMD increased significantly, by 27.4 ± 1.3 % after HDT (mean post-HDT value 57.7 ± 3.4 ms compared to post mean pre-HDT value 45.3 ± 2.1 ms; *p* < 0.05). The PMT decreased significantly by a mean of 21.4 % [pre 167.4 (SEM 10.1) ms compared to post 131.6 (SEM 16.2) ms; *p* < 0.01], and TRT decreased by a mean of 13.7 % [pre 157.3 (SEM 15.2) ms compared to post 135.8 (SEM 13.5) ms; *p* < 0.01)] after HDT.

The coefficients of correlation between the EMD and MVC before HDT were −0.67 (*p* = 0.05) and strongest correlations were found between EMD and ballistic voluntary force (*r* = −0.85; *p* = 0.01). The coefficients of correlation decreased between the EMD and MVC after HDT (*r* = −0.57; *p* = 0.05), and between the EMD and ballistic voluntary force (*r* = −0.70; *p* = 0.07).

## Discussion

Contractile properties of the skeletal muscles depend on integrity of the motor system and proprioceptive innervation and its prehistory which is crucial for maintaining the normal biological balance. Change in any of these factors calls for adaptive processes in muscle cell. The present investigation was focused on adaptive developments in the muscular apparatus following mechanical unloading by way of long-term bed-rest.

The major goal of the present study was to look into the effects of extended HDT on contractile properties and MTS of the TS in a group of young healthy women. The investigation demonstrated a significant alteration of the TS contractile properties as a result of the 120-day participation in the HDT experiment.

The present results confirm and extend our earlier data regarding the effects of short- (30) and long-term unloading (Koryak 1995, 2001) or disuse on the mechanical characteristics of a slow contracting muscle group such as the TS (Koryak 1984). Significant increases in maximal isometric twitch TPT and decreased 1/2 RT were maintained during a 120-day HDT. A likely explanation of the change in TPT in the disused limbs is a relatively greater atrophy of type I fibers (slow motor unit), which has been found to make up the majority of the TS (Johnson et al. 1973). However, since disuse produces muscle atrophy both in fast and slow skeletal muscles and, in addition, have been shown to cause fiber type-specific changes in the contractile properties (Gardetto et al. 1989), other factor(s) may be affected that alter fiber-type composition.

The rapid nature of the isometric changes, i.e., twitch duration, may be related to alterations in SR function (Briggs et al. 1977). The primary factor (mechanism) in the explanation of these changes may be a reduction in the rate at which Ca^2+^ is dissociated from the myofibrillar proteins (Briggs et al. 1977). Dissociation would occur more slowly if the rate of Ca^2+^ re-uptake by SR was decreased. Such a decrease has been found following disuse (Kim et al. 1982). A reduced rate of Ca^2+^ dissociation from myofibrillar proteins might be expected not only to increase the time course of the twitch response, but also to allow more force to be generated, since cross-bridges will continue to be formed while Ca^2+^ is available in the sarcoplasm. The tendency for *P*_t_ to decrease in this study cannot be explained. These effects on SR would be difficult to observe as the effects on *P*_t_ would be masked by atrophy, but are of interest on the assumption that the twitch changes are due to SR alterations. The changes in the kinetics of the mechanical responses to paired stimulation (cf. Fig. 3) might also be explained by altered development of Ca^2+^ kinetics in the muscles used in the experiment. The reduced twitch duration in the TS might in part result from lower *P*_t_ obtained in this muscle (cf. Fig. 2, top panel).

The decrease in *P*_t_ observed in these experiments after long-term HDT is in agreement with previous results which have shown a decrease in the maximal force during voluntary contractions and electrically evoked contractions (Koryak 1995, 2001, 2002). It was found that there was in all four subjects a proportionately identical decrease in *P*_t_ and in *P*_o_ at 150 impulses s^−1^. After HDT the electrically evoked contractions (*P*_o_) decreased significantly at a rate of 24 % of normal. *P*_o_ is a direct measure of the force-generating capacity of a muscle and has been considered to reflect the number of active interactions between actin and myosin (Close 1972). Disuse has been reported to produce decline in *P*_o_ by other workers (Duchateau and Hainaut 1990; Koryak 1995, 2001, 2002). This decline could reflect a decrease in the number of active cross-bridges and be expected to decrease the work capacity.

Two hypotheses may be suggested to account for the observation. First, the total number of cross-bridges could have been smaller after the period of disuse. Second, the force output per cross-bridge could have been decreased. However, Steven et al. (1990) have shown that when it was expressed per CSA, the force was unchanged after disuse. This would indicate that the first hypothesis of a decrease in the maximal number of cross-bridges was more appropriate to our results, rather than a change in density. Thus, the decline in the TS *P*_o_ could have been directly correlated with the decrease of the muscle fiber diameter and with muscle atrophy. The phenomenon of disuse as a factor in spaceflight or modeled microgravity is commonly related to losses in muscle volume/mass or CSA. However, most of the human muscles are pennate and, therefore, it would be more reasonable to interpret muscle atrophy and ensuing functional deviations with regard to alterations in the inner structure known as a muscle architecture.

The structural arrangement of muscle fibers within human skeletal muscle is an important factor contributing to the mechanical functioning of the muscle–tendon unit as a whole Previous studies in vivo using ultrasonography found that disuse instigates profound changes in muscular architecture, diminution of muscle fibers and reduction of their inclination compared with initial values, specifically (Friedrich and Brand 1990; Narici and Maganaris 2006; Koryak 2008). Reductions in muscle fascicle length and pennation angle reflect a loss of sarcomeres in-series, and in-parallel, respectively (Reeves et al. 2004; Narici and Maganaris 2006).

Of no less importance is the extent of change in tendon elasticity which determines length for the muscle fiber functioning (Reeves et al. 2004; Narici and Maganaris 2006) Maganaris et al. 2006) and, eventually, force-generating capacity of muscle. Moreover, restructuring of the MTC also may contribute considerably to the contraction force decrement (Kubo et al. 2004). The relationship between muscle fiber length and inclination is highly specific for a muscle. Architecture and its internal properties are the major determinants of muscle function (Kawakami et al. 2001). In supine position with the knee at 180° (full extension), the ankle assumes a slight plantar curvature. It should be noted that displacement of body segments in the course of unloading was confirmed by our earlier data (Clément et al. 1985) according to which in real microgravity the human adopts a specific, fetal posture that presupposes changes of the ankle joint position and, consequently, length of the plantaris extensors. In the present investigation, this induced stimulus of muscle physiological shortening was probably sufficient to contribute to the reductions of myofibers length and inclination, as well as muscle thickness, which is in accord with the findings in simulation studies (Kawakami et al. 2001; Kubo et al. 2004).

In addition, the decline in *P*_o_ of the whole muscle would suggest that long-term 5° HDT may deleteriously affect one of the steps in excitation–contraction coupling (Duchateau and Hainaut 1990; Koryak 2002). Possibilities include alterations in the sarcolemma action potential, the T-tubular charge movement, and/or direct effects on the SR Ca^2+^ release channel. Alternatively, the disuse-induced muscle atrophy may enlarge the extracellular space such that the tension per whole muscle decreases more than per fiber CSA.

The mechanisms responsible for the loss of force with disuse are not well understood, but they cannot include decreases in the CSA of slow- and fast-twitch muscle fibers of the muscle. Muscle atrophy, therefore, probably contributed to the loss of force (Dudley et al. 1992). Morphological analyses were not performed in the present study. Hikida et al. (1989) have shown that relative changes in muscle and fiber sizes were less than the relative change in force and that changes in the ultra-structure may diminish the force output ability of skeletal muscle during and following long-term exposure to microgravity.

The much larger (36 %) reduction in MVC when compared to the insignificant changes in *P*_o_ after a 120-day HDT (24 %) may indicate an inability of the central nervous system to activate the TS normally. Whether this was due to a lack of motivation on the part of the subjects, or to an involuntary reduction in neural drive, is difficult to distinguish. The subjects certainly appeared well motivated and had no discomfort or knee stiffness before performing the test which could have accounted for the low MVC. The increase in force deficiency (see Fig. 1, top panel) would suggest a decline in central drive in the control of voluntary muscle by the motor nerve system. In fact, during MVC, the electromyogram activity has been found to be significantly changed by inactivity itself (Duchateau and Hainaut 1990; Recktenwald et al. 1999). Moreover, observation of amplitude changes after inactivity has suggested that fewer motor units were activated in disused muscle (Duchateau and Hainaut 1990), and maximal firing frequency of motor unit has been found to be decreased (Mayer et al. 1981). It has been thought that a decrease in maximal firing rate could be explained by changes in proprioceptive afferents on the motoneurons (Mayer et al. 1981). This suggests that in future studies in humans cognizance must be taken of the initial physiological states of the muscles that are to be disused to access the extent to which neural and muscle function is affected by loss of voluntary movement.

The rate of rise of evoked contraction in response to electrical stimulation of the nerve with a frequency of 150 impulses s^−1^ calculated according to a relative scale changed very little due to HDT. This observation agrees with the data obtained earlier (Witzmann et al. 1982; Koryak 2001). Witzmann et al. (1982) have shown that there were no significant changes in the force–velocity characteristics of rat soleus, extensor digitorum longus or superior, and vastus lateralis muscles after 21-days of immobilization, or in the human TS after 120 days of HDT (Koryak 1995, 2001) and is consistent with the observed relative constancy of the mechanics of the tetanus and current (cross-bridge) theories of muscle contraction (Ranatunga 1982). It would therefore seem reasonable to conclude that disuse, for example HDT, in women patients has little effect on either cross-bridge cycling or myosin activity (Close 1972).

The investigation provided first data about EMD alterations during voluntary the TS contractions in the group of females following long-term HDT. As it is known, EMD is a peripheral component of human motor reaction embracing the lag from the onset of muscle-agonist EMG till actual motion or, in other words, time of stretching the series viscoelastic component by the contractile elements (Cavanagh and Komi 1979), which, in its turn, is dependent on force generation rate (Cavanagh and Komi 1979; Thomason and Booth 1990). Consequently, EMD increase/reduction can be an indirect indicator of changed MTC stiffness (Mora et al. 2003).

The present study has shown that EMD response to long-term HDT suggests changes in the TS properties. Previous results demonstrated convincingly that unloading can alter mechanic behavior of the muscle tendon, and that tendon extension results in decrease of tendon stiffness (Mayer et al. 1981; Mora et al. 2003; Kubo et al. 2000, 2004; De Boer et al. 2007). This loss in tendon stiffness may amplify its deformation in the course of force generation. As a result, muscle fibers shift in the nonoptimal zone of the tension–length relation. Earlier it was shown that the greatest contraction force is determined on the tension–length plateau (Ichinose et al. 1987; Recktenwald et al. 1999). Therefore, exaggerated shortening of contracting muscle fibers due to increased tendon deformation makes sarcomeres work at shortened, far from optimal, lengths, and consequent lower force production.

Changes in muscle MTC stiffness influence the rate of contractile force transfer to the bone system. In this investigation, we explored two variables associated with the rate of force transfer from muscle to the skeleton (i.e., the time required to transmit contraction forces to bones and rate of tension rise). The latter is dependent on tendon stiffness and contraction rate at which force is transferred to the bone system, whereas EMD is dependent on the propagation of action potential along on muscle membrane, the excitation–contraction coupling processes, and the stretching of the series elastic component by the contractile element (Wilkie 1949). Since it is known that unloading inhibits the rate of excitation transfer along the membrane of any type of muscle fiber (4), this may contribute to EMD prolongation. According to our data, post-HDT EMD increased (+27.4 %) with reduction of the voluntary contraction rate suggesting a significant prolongation of the time of communication between excitation–contraction and viscoelastic series components which can be a result of tendon stiffness reduction. These data are in good agreement with data in Kubo et al. (2004). It should be noted that EMD prolongs substantially in consequence of gross loss in tendon stiffness (Costa et al. 2010), however, EMD does not alter in the event when “weak tendon is raised” (Muraoka et al. 2004) and extends the MTC (Mora et al. 2003).

As shown in this study EMD finds a negative relationship with the MVC; i.e., shorter the EMDs, the higher the MVC. This means that subjects with higher MVC have a high content of fiber type II. Interestingly, this relationship varies with the experimental conditions. So, after unloading there is a weakening of the connection; in other words, there is less dependence on the proportion of EMD type II fibers in the muscle. This may be due to differences in recruitment strategy motor unit, i.e., less type II fibers active at a lower rate of force development and therefore a lesser dependence of the EMD on the proportion of this type of fibers in the muscle These data are comparable with previous studies that found a negative correlation between EMD and in fiber type II (Norman and Komi 1976; Nilsson et al. 1977). Viitasalo and Komi (1981) have noted negative correlations between MVC and EMD, between the proportion of type II fibers and EMD, and a positive correlation between the proportion of type II fibers and MVC (Komi and Tesch 1979).

The conclusion, the results of the reported investigation demonstrated changes in the mechanical properties of human the TS in consequence of long-term HDT. Comparison of mechanical parameters registered during voluntary and electrically evoked contractions infers that the experimental conditions affect not only the peripheral processes of contraction but also nervous (cortex) motor control of maximal voluntary contraction, so that reduced frequency of already recruited motor units or decrease the number of recruited motor units, or both ways. Moreover, the investigation demonstrates a direct correlation between EMD and changed MTS post-HDT. Thus, EMD can be an indirect index of the extent of change in muscle MTC stiffness.

## Abbreviations

CSA: Cross-sectional area
EMD: Electromechanical delay
EMG: Electromyography
HDT: Head-down tilt
1/2 RT: Half-relaxation time
MT: Motor time
MTC: Muscle–tendon complex
MTS: Musculo-tendinous stiffness
MVC: Maximal voluntary contraction
PMT: Premotor time
*P*_d_: Force deficiency
*P*_o_: Maximal force
*P*_t_: Twitch force
SR: Sarcoplasmic reticulum
TI: Tetanic index
TPT: Time-to-peak
TRT: Total reaction time
TS: Triceps surae
